# Patch-to-Seq and Transcriptomic Analyses Yield Molecular Markers of Functionally Distinct Brainstem Serotonin Neurons

**DOI:** 10.3389/fnsyn.2022.910820

**Published:** 2022-06-30

**Authors:** Gary C. Mouradian, Pengyuan Liu, Pablo Nakagawa, Erin Duffy, Javier Gomez Vargas, Kirthikaa Balapattabi, Justin L. Grobe, Curt D. Sigmund, Matthew R. Hodges

**Affiliations:** ^1^Department of Physiology, Medical College of Wisconsin, Milwaukee, WI, United States; ^2^Neuroscience Research Center, Medical College of Wisconsin, Milwaukee, WI, United States; ^3^Cardiovascular Center, Medical College of Wisconsin, Milwaukee, WI, United States; ^4^Department of Biomedical Engineering, Medical College of Wisconsin, Milwaukee, WI, United States; ^5^Comprehensive Rodent Metabolic Phenotyping Core, Medical College of Wisconsin, Milwaukee, WI, United States

**Keywords:** CO_2_ chemoreception, 5-HT neurons, patch-seq, PH sensitivity, brainstem 5-HT, raphe magnus, transcriptomic analysis, medullary raphe

## Abstract

Acute regulation of CO_2_ and pH homeostasis requires sensory feedback from peripheral (carotid body) and central (central) CO_2_/pH sensitive cells – so called respiratory chemoreceptors. Subsets of brainstem serotonin (5-HT) neurons in the medullary raphe are CO_2_ sensitive or insensitive based on differences in embryonic origin, suggesting these functionally distinct subpopulations may have unique transcriptional profiles. Here, we used Patch-to-Seq to determine if the CO_2_ responses in brainstem 5-HT neurons could be correlated to unique transcriptional profiles and/or unique molecular markers and pathways. First, firing rate changes with hypercapnic acidosis were measured in fluorescently labeled 5-HT neurons in acute brainstem slices from transgenic, Dahl SS (SSMcwi) rats expressing T2/ePet-eGFP transgene in Pet-1 expressing (serotonin) neurons (SS^*ePet*1–eGFP^ rats). Subsequently, the transcriptomic and pathway profiles of CO_2_ sensitive and insensitive 5-HT neurons were determined and compared by single cell RNA (scRNAseq) and bioinformatic analyses. Low baseline firing rates were a distinguishing feature of CO_2_ sensitive 5-HT neurons. scRNAseq of these recorded neurons revealed 166 differentially expressed genes among CO_2_ sensitive and insensitive 5-HT neurons. Pathway analyses yielded novel predicted upstream regulators, including the transcription factor *Egr2* and *Leptin*. Additional bioinformatic analyses identified 6 candidate gene markers of CO_2_ sensitive 5-HT neurons, and 2 selected candidate genes (*CD46* and *Iba57*) were both expressed in 5-HT neurons determined via *in situ* mRNA hybridization. Together, these data provide novel insights into the transcriptional control of cellular chemoreception and provide unbiased candidate gene markers of CO_2_ sensitive 5-HT neurons. Methodologically, these data highlight the utility of the patch-to-seq technique in enabling the linkage of gene expression to specific functions, like CO_2_ chemoreception, in a single cell to identify potential mechanisms underlying functional differences in otherwise similar cell types.

## Introduction

The neural network coordinating respiratory muscle activity dynamically maintains blood gas homeostasis via modulation from hypoxic (low O_2_) and hypercapnic (high CO_2_) ventilatory chemoreflexes. Central respiratory chemoreceptor neurons in the hindbrain may encode local CO_2_/pH levels and adjust ventilatory drive accordingly. These are central respiratory chemoreceptor cells (neurons and/or glia) and include distinct subpopulations of phox2b^+^ glutamatergic neurons in the retrotrapezoid nucleus (RTN) and Pet-1^+^ serotonergic (5-HT) neurons. While still a topic of debate (Corcoran et al., 2009), CO_2_/pH responses in these hindbrain neurons are considered unique and intrinsic properties, where acidification increases firing rates independent of synaptic inputs to encode CO_2_/pH status and modulate breathing. Data continue to emerge regarding the mechanisms underlying cellular sensing of CO_2_/pH and associated changes to firing rate in RTN neurons, which appear to require the expression of a pH-sensitive potassium ion channel (TASK-2), G protein-coupled receptor 4 (Gpr4) (Kumar et al., 2015) and 5-HT receptor activation (Wu et al., 2019). Local RTN astrocytes may instead rely on pH-sensitive inwardly rectifying potassium ion channels (Kir4.1 and/or Kir5.1) to sense changes in local milieu CO_2_/pH levels (Gourine et al., 2010; Wenker et al., 2010). Less progress has been made in identifying molecular mechanisms of CO_2_ sensitivity in 5-HT neurons, but it appears unlikely that they rely on the same mechanisms as RTN neurons (Mulkey et al., 2007; Teran et al., 2014).

Whereas midbrain 5-HT neurons critically modulate higher brain functions such as sleep-wake cycles (Buchanan et al., 2008; Cui et al., 2016), hindbrain 5-HT neurons are critical for vital physiological functions including respiratory control (Feldman et al., 2003; Hodges and Richerson, 2008; Ptak et al., 2009). In addition to their pH/CO_2_ chemosensitive properties, these anatomically caudal groups of 5-HT neurons modulate ventilatory output *via* tonic release of excitatory 5-HT and co-released neuropeptides, substance P and thyrotropin-releasing hormone (Hodges and Richerson, 2008; Corcoran et al., 2009). Dysfunction of the brainstem 5-HT system is linked to the unexpected respiratory failure in Sudden Infant Death Syndrome (SIDS) and it may be linked to Sudden Unexpected Death in Epilepsy (SUDEP) (Buchanan, 2019). Thus, characterization of the functional contributions of distinct subsets of hindbrain 5-HT neurons (modulatory vs. CO_2_ sensitive) may lead to a better understanding of the pathogenesis of these devastating outcomes.

Subpopulations of brainstem 5-HT neurons increase their firing rates in response to hypercapnic acidosis, and experimentally induced dysfunction of 5-HT neurons reduces hypercapnic ventilatory responses *in vivo* (Hodges et al., 2007, 2008; Wu et al., 2008; Corcoran et al., 2009; Ray et al., 2011; Kaplan et al., 2016; Cerpa et al., 2017). Intersectional fate mapping in mice demonstrates that embryonic origins of pH-sensitive 5-HT neurons are rhombomere (R)5 (Pet1- and Egr2-expressing cell lineage)-specific (Brust et al., 2014), whereas other subpopulations of brainstem 5-HT neurons (R6 or Tac1/Pet1-expressing) are not CO_2_ sensitive but likely modulate respiratory motor outputs. All but rhombomere 4 5-HT neurons express Pet1 during development (Hendricks et al., 1999). These data indicate that subpopulations of hindbrain 5-HT neurons programmed during embryonic development for distinct post-natal functional contributions to ventilatory control. They also suggest CO_2_ sensitive 5-HT neurons express distinct sets of genes which may be determinants and/or biomarkers of their unique CO_2_ sensing properties. However, it remains unknown if postnatal phenotypically defined 5-HT neurons have similar transcriptional differences as 5-HT neurons defined by embryonic origin.

**FIGURE 1:**
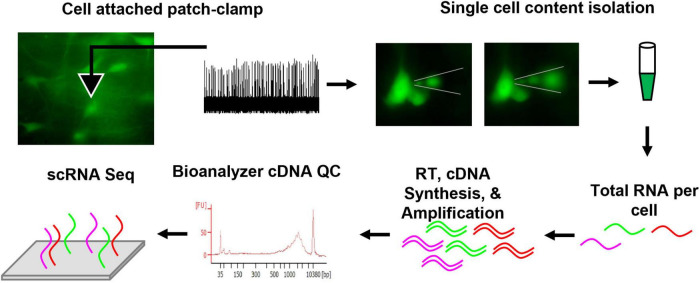
Methodological schematic outlining the patch-seq technique. In summary, eGFP 5-HT neurons were fluorescently identified from an acute brainstem slice, loose cell attached patch-clamp yielded firing rate measurements during control, CO_2_ challenge, and washout periods. Thereafter, the recorded neuron’s cell contents were isolated under fluorescence to visualize removal of intracellular content. The content was collected in sterile PBS and frozen. Total RNA per cell was reverse transcribed (RT), cDNA was synthesized, and amplified. Quality control (QC) was completed using an Agilent Bioanalyzer. Samples passing QC were submitted for sequencing. Further methodological details can be found in the section “Materials and Methods”.

Here, the patch-to-seq technique was employed (Figure 1; Cadwell et al., 2016; Chen et al., 2016; Fuzik et al., 2016; van den Hurk et al., 2018) to determine if postnatal cellular CO_2_ responses in brainstem 5-HT neurons could be attributed to unique transcriptional profiles. In acute brainstem slice preparations from transgenic rats expressing GFP in all serotoninergic neurons, 5-HT neuronal firing rate was measured during control and hypercapnic acidosis using cell-attached patch clamp electrophysiology. The rat pups were derived from the Dahl SS (SSMcwi) strain which have a robust CO_2_ response by 18 days of age (Davis et al., 2006) and expressed the T2/ePet-eGFP transgene to direct enhanced GFP expression in Pet-1-expressing (serotonin) neurons (SS^*ePet*1: eGFP^; RGD strain ID: 8661234). Subsequently, intracellular contents from the recorded neurons were collected for single cell RNA sequencing (scRNA-Seq; not to be confused with 10x genomics) and subsequent transcriptomic analyses. Combining these techniques provides a powerful approach for correlating electrophysiological responses to CO_2_/pH changes with transcriptional data on a cell-to-cell basis.

## Materials and Methods

### Animals

SS^*ePet*1: eGFP^ rats (*n* = 72) that have restricted expression of eGFP to all CNS 5-HT neurons as previously described (Katter et al., 2013) were used between 18 and 23 days of age. Male and female rats were housed in the Biomedical Resource Center when not undergoing study with a standard 12:12 h light/dark cycle. Rats had *ad libitum* access to food (Dyets Inc., Bethlehem, PA, United States, 0.4% salt: #113755) and hyperchlorinated water. All experiments were approved by the Medical College of Wisconsin Institutional Animal Care and Use Committee prior to experimental use.

### Electrophysiological Recordings in Brainstem Slices

Rat pups (P18-23) were anesthetized with 2.5% isoflurane and transcardially perfused with 5 mL of NMDG solution (in mM: 2.5 KCl, 1.25 NaH_2_PO_4_, 26 NaHCO_3_, 0.5 CaCl_2_-2H_2_0, 7 MgSO_4_-7H_2_0, 92 NMDG, 20 HEPES, 25 glucose, 2 thiourea, 5 Na-ascorbate, and 3 Na-pyruvate at 7.5 adjusted pH). The brain was quickly isolated and placed into NMDG solution. The first quarter of the frontal brain was grossly cut and then the brain was embedded in 4 mL of 0.04 g Agarose (Sigma-Aldrich, Burlington, MA, United States; A0576) dissolved in 0.9% NaCl solution. Coronal sections 200 μm thick were made caudal to rostral using a vibratome (Leica; VT 1200S) in room temperature bubbled NMDG solution. Brainstem slices were then incubated for 12 min in heated (32°C) NMDG solution and then for 10 min in heated (32°C) normal aCSF solution (in mM: 3.0 KCl, 2.0 CaCl_2_-2H_2_O, 1.0 MgCl_2_-6H_2_0, 1.25 NaH_2_PO_4_, 123 NaCl, 25 NaHCO_3_, 10 glucose) before incubating at room temperature normal aCSF solution for 1 h prior to commencing electrophysiology recordings. All solutions were bubbled with 95% O_2_ and 5% CO_2_).

All working surfaces including the microscope, objectives, micromanipulators, pipette puller, beakers, and computer workstation were cleaned with DNA-OFF (Takara, San Jose, CA, United States; #9036) and RNase Zap (Life Technologies, San Jose, CA, United States; #AM9780). Then, brainstem slices inclusive of raphe magnus (approximately −12 to −10 mm from bregma) were placed in the recording chamber on a fixed-stage fluorescent microscope (Nikon; Eclipse FN1) equipped with DIC optics (Photometrics; CoolSNAP ES2). 5-HT neurons targeted for recording and single cell content isolation were identified by positive eGFP fluorescence.

Neuron recordings (*n* = 91) were performed using loose-cell attached patch-clamp recordings at a bath temperature of 37°C using pCLAMP recording software connected to a MultiClamp amplifier and a Digidata 1440A analog-to-digital converter (each from Molecular Devices). Patch clamp electrodes (2.5–6 MΩ) were backfilled with normal aCSF. Normal aCSF spiked with synaptic blockers was used in the perfused bath for recordings [in μM: 10 CNQX (Abcam; ab120017), 50 D-AP-5 (Abcam; ab120003), and 10 Gabazine (Abcam; ab120042)]. Firing rate was measured during control conditions (95% O_2_/5% CO_2_; pH: 7.363) for 5 min then during a hypercapnic challenge (85% O_2_/15% CO_2_; pH 7.092) for 10 min, followed by at least 10 min upon returning to control conditions. Bath pH was sampled by syringe and measured on a blood gas analyzer (Radiometer; ABL800 FLEX). The chemosensitivity index (C.I.) was calculated as the average for each of the two condition changes per experiment (from baseline to CO_2_ challenge and from CO_2_ challenge to recovery) using the following formula adapted from a prior report (Wang et al., 1998).

C.I.=100%x 10(log⁡(F⁢R⁢15)-log⁡(F⁢R⁢5)(p⁢H⁢5-p⁢H⁢15)/0.2)             

Where FR15 is the average firing rate at 15% CO_2_ challenge, FR5 is the average firing rate at 5% CO_2_ conditions (control and recovery), pH5 is the average pH at 5% CO_2_, and pH15 is the average pH at 15% CO_2_. The logarithms are in base 10. The 0.2 constant represents the 0.2 pH unit changes used to initially develop this formula as detailed previously (Wang et al., 1998). The C.I. was used to determine which 5-HT neurons were chemosensitive. Following cell recordings, a second micropipette was backfilled with <1 μl of modified normal aCSF [in mM unless stated otherwise: 123 K-gluconate, 12 KCl, 10 HEPES, 0.20 EGTA, 4 MgATP, 0.30 NaGTP, 10 Na-phosphocreatine, and 1U/μl RNAse inhibitor (40U/μl; Takara, San Jose, CA, United States; #2313A)] based on published Patch-Seq methods (Cadwell et al., 2016). Note, we omitted glycogen addition as it interfered with cDNA synthesis. The backfilled micropipette was advanced to the recorded neuron using slight positive pressure. Positive pressure was released upon nearing the recorded neuron (verified by location of recording pipette). Then, negative pressure was applied to isolate intracellular content monitored under live fluorescence imaging to visualize movement of eGFP+ content into the isolation micropipette (and shrinking of neuron). Fluorescent images before and after the isolation were obtained for documentation (Figure 1). The isolation pipette was quickly removed from the tissue and the tip broken inside a sterile RNAse/DNAse free 0.5 mL microcentrifuge tube and intracellular content was gently aspirated into 4 μl of sterile PBS (No Ca^2+^ no Mg^2+^). The sample was then flash frozen on dry ice and stored at −80°C until cDNA creation. Less than 3 min elapsed from isolation to flash freezing to minimize RNA degradation.

### Library Construction

We converted total RNA to complementary DNA (cDNA) using the SMART-Seq v4 Ultra Low Input RNA Kit and associated protocol (Takara Bio USA, Inc., San Jose, CA, United States, #634890). Briefly, 4 μl of isolated sample was thawed on ice, spun down, and transferred into 0.2 mL RNAse/DNAse free PCR tubes. To begin first strand cDNA synthesis, volume was brought to 9.5 μl with nuclease-free water. 1 μl of 10x reaction buffer was added to each tube before adding 1 μl of 3′ SMART-Seq CDS Primer IIA. Samples were mixed well and spun down prior to incubating the tubes at 72°C in a preheated, hot-lid thermal cycler for 3 min and then immediately placed on ice for 2 min. Then, 7.5 μl of master mix, containing 5X Ultra Low First-Strand Buffer, SMART-Seq v4 Oligonucleotide, RNase Inhibitor, and SMARTScribe Reverse Transcriptase, was added to each sample for completion of first strand cDNA synthesis using the outlined thermal cycler program indicated in the protocol. Next, cDNA was amplified using 17 PCR cycles after the addition of PCR Master Mix (30 μl) containing 2X SeqAmp PCR Buffer, PCR Primer II A, and SeqAmp DNA Polymerase, was added to each sample. The number of PCR cycles was determined based on protocol recommendation and from earlier pilot experiments. The amplified cDNA was then purified using immobilization based clean up with Agencourt AMPure XP beads (Beckman Coulter Inc., Brea, CA, United States, #A63880) and fresh 80% ethanol. cDNA was then eluted (17 μl) with the Elution Buffer and transferred into new PCR tubes. An Agilent 2100 Bioanalyzer and an Agilent High Sensitivity DNA Kit (Agilent, Santa Clara, CA, United States; #5067-4626) was used to validate success of cDNA synthesis and amplification. All sequenced samples yielded a peak at ∼2,500 bp that spanned over 400 and 10,000 bp with over 3.5 ng of cDNA. Negative control samples yielded no peaks. cDNA library preparation was completed on validated cDNA samples using Illumina Library preparation protocols outlined in the NextEra XT DNA Library Preparation Kit (Illumina, Madison, WI, United States; #FC-131-1024). As suggested by the Takara SMARTER-Seq v4 protocol, 100–150 pg of amplified cDNA was used as the input volume for the NextEra XT DNA Library Preparation Kit. Libraries were fragmented and tagmented with adapters for sequencing according to the NextEra XT Index Kit (Illumina, Madison, WI, United States; #FC-131-2001). cDNA library sizes (250–1,500 base pairs) and concentration (>2 nM) were verified prior to sequencing using the Agilent High Sensitivity DNA Kit.

### RNA Sequencing

Paired end sequencing was completed on an Illumina HiSeq platform in-house. We performed sequence data analyses using established in-house pipelines for read mapping, alignment, transcript quantification, and statistical differential expression (Mouradian et al., 2016) and DESeq2 (Love et al., 2014) for batch-effect normalization of data obtained from two separate flow-cells. Briefly, the Tuxedo Suite was used for RNA Sequencing analysis. The Bowtie and TopHat v2 software components were used for sequence alignment, and Cufflinks for assembling reads into transcripts and differential gene expression using the Cuffdiff algorithm.

### RNA Sequencing Analyses

#### Hierarchical Clustering

Hierarchical clustering was performed using the heatmap.2 function of the gplots R package as an alternative visualization method to multidimensional scaling by calculating Euclidean distances from logFPKM of the top 100 most variable genes. The heatmap.2 function produced a false colored image based on the top 100 most variable genes and attached a dendrogram based on the default parameters of the heatmap.2 function^1^.

#### Multidimensional Scaling

Multidimensional scaling was performed and plotted using the R package limma using the plotMDS function to produce a principal coordinate plot which was used to visualize the distances between each sample and their gene expression profiles represented by the columns of *x* (Ritchie et al., 2015; Law et al., 2016). Samples are plotted on a two-dimensional scatterplot so that the distances on the plot approximate the typical log2 fold change between samples. The first dimension represents the leading-fold change that best separates samples and explains the greatest proportion of variation in the dataset. The subsequent plotted dimensions have a smaller effect and are orthogonal to the ones plotted prior.

#### Support Vector Machine-Recursive Feature Elimination

Traditional support vector machine (SVM) learning with R packages such as “e1071” can be too noisy because there are too many genes, and the method is computationally intensive. Our study utilized the R package pathClass to narrow down the number of genes relevant to the outcome, which makes SVM cleaner and less computationally intensive. This method is known as SVM recursive feature elimination (SVM-RFE), which builds a model based on the current number of genes and removes a gene of interest to then check the accuracy of the model. Additionally, “e1071” was used to implement SVM to build a model to see what genes are important in prediction chemosensitivity. First, SVM-RFE was implemented using pathClass. PathClass reduced the number of genes used as predictors for the outcome (CO_2_ sensitive vs. insensitive) to reduce intensive computation and eliminate overfitting of data, a consequence too many predictors. After predictors were identified by pathClass, “e1071” was used to implement SVM to build a model using the predictor genes to visualize what genes are important in predicting chemosensitivity and to what accuracy. A radial basis function was used for the kernel (a kernel is simply a shape in high-dimensional space used to segregate the data [e.g., points (cells)] that fall inside or outside a circular shape in the case of a radial basis function). Four tests were performed to predict accuracy and for each test one predictor gene was used and seven random samples were left out of the analysis to function as a test group while the remaining samples (Kaplan et al., 2016) were processed as training data. The accuracy of the proposed model was tested to ensure that cells were classified accurately. The model used was: svm.model <- svm(Labels ∼ data = trainset, cost = 100, gamma = 1). Next, the model was looped 10,000 times to see how accurate the radial kernel SVM (predicting only based on one gene at a time due to large list of predictors) is in the long run. After the 10,000 trials, the radial kernel SVM was used to predict the percentage of accuracy. The next tests performed evaluated how altering the value of C (cost) affected the accuracy of the classifier. C was changed to 1,000 (high cost) and 10 (low cost). Again, after 10,000 trails looking at low and high cost, the radial kernel SVM was used to predict the percentage of accuracy. Finally, a polynomial kernel was used with 100,000 trials using the same model. SVM-RFE iteratively eliminated features that are less significant in determining the classification model (i.e., the gene or list of genes most able to predict if a 5-HT neuron is CO_2_ sensitive or not (Huang et al., 2014).

#### RNAScope

Brains were collected after transcardial perfusion with Dulbecco’s phosphate-buffered saline (DPBS, Gibco) containing 0.1% heparin followed by 4% paraformaldehyde (PFA, 4% in PBS, Biotium). The brains were dissected and post fixed in 4% PFA for 24 h and dehydrated with serial, 24-h sucrose solutions (10, 20, then 30% sucrose). The tissue was then place into a cryomold with Tissue-Tek optimal cutting temperature compound (O.C.T., Sakura) and snap frozen in dry ice and 4-methylbutane. Tissue was cryosectioned at 14 μM and stored at −80°C until use. Endogenous mRNA was detected using the *in situ* hybridization technique RNAscope^®^ 2.5 High Definition – Fluorescent Multiplex Assay (Wang et al., 2012). Briefly, tissues were placed in 10% neutral buffered formalin (10% NBF, Sigma-Aldrich, Burlington, MA, United States) for 15 min at 4°C, dehydrated in a serial ethanol dilution up to 100% ethanol before blocking endogenous peroxidase activity with H_2_O_2_ and treatment with protease plus (ACD Bio). Slides were then incubated for 2 h at 40°C with probes identifying tryptophan hydroxylase 2 (*Tph2*; Rn-Tph2 – 316411, ACD Bio), *CD46* (*CD46*; Rn-*CD46*-C2 – 847661-C2, ACD Bio), Iron-Sulfur Cluster Assembly Factor *IBA57* (*Iba57;* Rn-*Iba57*-C2 – 847651-C2, ACD Bio) or positive control probe peptidylprolyl isomerase B (*Ppib*; Mm-Ppib – 313911, ACD Bio), RNA polymerase II subunit A (Mm-Polr2a-C2 – 312471-C2, ACD Bio), and negative control probe 4-hydroxy-tetrahydrodipicolinate reductase (*dapB;* 310043, ACD Bio). RNAscope^®^ fluorescent multiplex assay protocol was followed for all other amplifications and development steps. Slides were stained with DAPI and cover slipped with ProLong Gold (P10144, Thermo Fisher Scientific, Coraopolis, PA, United States).

#### RNAScope Analysis

Max projection images were acquired by a blinded investigator from 5 fluorescent *z*-sections (each 2 μm apart) from tissue at 4 and 20× magnification via a Nikon A1 confocal microscope using NIS elements software. Imaging settings (laser power, gain, offset, and photomultiplier tube) were consistent throughout the experiment. Images were loaded in ImageJ (FIJI) for analysis (Schindelin et al., 2012). Each Tph2+ cell was manually outlined. Tph2 images were autothresholded while all *CD46* and *Iba57* images were thresholded at 9 and 10 on a 255 white-black scale, respectively. Then, the Tph2+ cell outlines were overlaid onto the thresholded *CD46* or *Iba57* images to count the number of Tph2+ cells expressing *CD46* or *Iba57* and the total number of Tph2+ cells. A similar quantification was repeated within the raphe magnus outlined guided by the rat brain atlas (Paxinos and Watson, 1998). Samples were then unmasked and identified. Data are reported as *CD46* or *Iba57* as a percentage of Tph2 + cells, either throughout the section or within the raphe magnus.

#### Statistical Analyses

Statistical analyses of data were performed using Prism (GraphPad Prism 9.0, GraphPad Software, LLC, San Diego, CO, United States). Data are reported as mean ± SEM unless otherwise specified. Differences between groups in electrophysiological parameters were detected using an unpaired Student’s *t*-test or across conditions with one- or two-way Repeated Measures ANOVA with Sidak’s *post hoc* tests using log normalized values to achieve parametric assumptions. Un-normalized electrophysiology data are reported in the section “Results.” Simple linear regressions were used to determine correlations between gene expression and a given electrophysiologic metric. Samples were determined outliers if its value was greater than two standard deviations from the mean. Alpha (*p*-value) < 0.05 is statistically significant.

## Results

### Functional Segregation of 5-HT Neurons Based on CO_2_/pH Sensitivity

**FIGURE 2:**
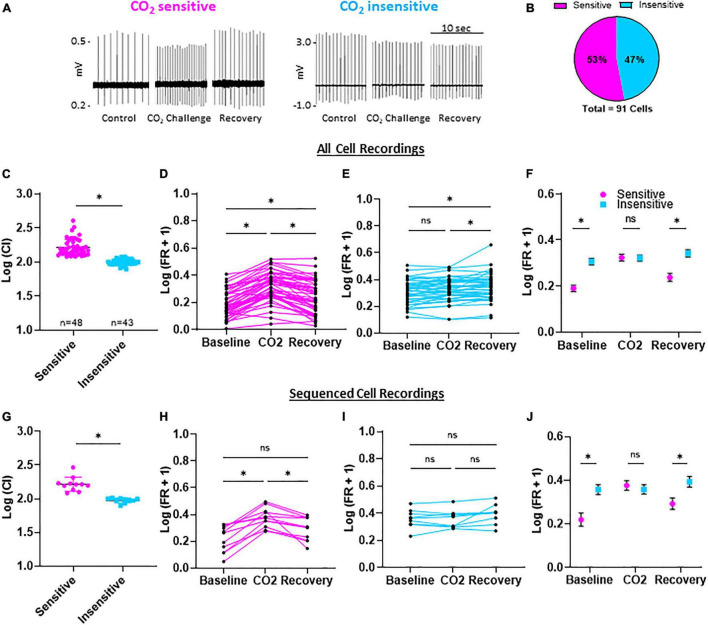
Firing rates and chemosensitivity index (C.I.) of CO_2_ sensitive and insensitive 5-HT neurons. Representative, condensed recording traces during control (95% O_2_/5% CO_2_; 7.363 pH), CO_2_ challenge (85% O_2_/15% CO_2_; 7.092 pH), and recovery (95% O_2_/5% CO_2_; 7.363 pH) **(A)**. Distribution of CO_2_ sensitive or insensitive 5-HT neurons among 91 neurons **(B)**. Log transformed chemosensitivity index (C.I.) of CO_2_ sensitive and insensitive 5-HT neurons **(C)**. Log transformed firing rates during baseline, CO_2_ challenge, and recovery compared among all CO_2_ sensitive **(D)**, CO_2_ insensitive **(E)**, and between CO_2_ sensitive and insensitive **(F)** 5-HT neurons. The sub-population of neurons used for scRNA-Seq had representative log transformed C.I. values, and firing rate differences during baseline, CO_2_ challenges, and recovery **(G–J)**. Notably, baseline and recovery firing rates were lower in CO_2_ sensitive vs. insensitive 5-HT neurons while firing rate during the CO_2_ challenge was similar between CO_2_ sensitive and insensitive neurons. Unpaired, two-tailed student’s *t*-test **(C,G)**. One-way repeated measures ANOVA with Sidak’s multiple comparisons test **(D,E,H,I)**. ANOVA results: Condition *p* < 0.0001 and Individual Cell *p* < 0.001 **(D)**; Condition and Individual Cell *p* < 0.0001 **(E)**; Condition *p* = 0.0002 and Individual Cell *p* = 0.0025 **(H)**; Condition *p* = 0.0539 and Individual Cell *p* < 0.0001 **(I)**. Two-way repeated measures ANOVA with Sidak’s multiple comparisons test **(F,J)**. Condition *p* < 0.0001, Cell Phenotype *p* = 0.0005, and Interaction *p* < 0.0001 **(F)**; Condition *p* = 0.0002, Cell Phenotype *p* = 0.0279, and Interaction *p* < 0.0001 **(J)**. *p < 0.05 and ns, not significant.

All eGFP-expressing 5-HT neurons showed spontaneous action potentials under control conditions (5% CO_2_, pH = 7.363), which ensured cell viability and membrane integrity in the cell-attached configuration (Figure 2A). Switching superfusate to CO_2_-enriched aCSF (15% CO_2_, pH = 7.092) reversibly increased firing rates (FRs) in some eGFP + 5-HT neurons. Using a calculated chemosensitivity index (C.I.; see section “Materials and Methods”), each of the 91 5-HT neurons recorded were classified as CO_2_ sensitive (C.I. >120; *n* = 48) or CO_2_ insensitive (C.I. <120; *n* = 43; Figure 2B). The average C.I. for CO_2_ sensitive 5-HT neurons was 169.8 ± 8.3 (vs. 100.1 ± 1.4 for insensitive; *P* < 0.0001; Figure 2C). FRs in individual CO_2_ sensitive 5-HT neurons significantly increased during the challenge and then decreased upon washout (recovery; Figure 2D) but remained slightly higher than control (*P* < 0.0001 for baseline FR vs. CO_2_ challenge; *P* = 0.0004 for baseline vs. recovery). In contrast, FRs in CO_2_ insensitive 5-HT neurons changed little during the CO_2_ challenge and in recovery (Figure 2E; *P* > 0.05 for baseline FR vs. CO_2_ challenge; *P* = 0.0092 for CO_2_ challenge vs. recovery). CO_2_ sensitive and insensitive 5-HT neurons differed in their responses to hypercapnic acidosis and had distinct basal FRs which were lower in CO_2_ sensitive 5-HT neurons vs. insensitive 5-HT neurons at baseline (*P* = 0.0012) and recovery conditions (Figure 2F; *P* = 0.0010). There were no FR differences between CO_2_ sensitive and CO_2_ insensitive 5-HT neurons during the CO_2_ challenge (*P* = 0.3475). See Supplementary Figures 1A–F for raw firing rate values per condition per neuron. Such distinct basal FRs are highly predictive of 5-HT neuron chemosensitivity (Supplementary Figures 1G–J). Among the 91 neurons recorded, 21 neurons (*n* = 11 CO_2_ sensitive; *n* = 10 CO_2_ insensitive) yielded sufficient RNA of high quality for manual, not 10x Genomics, scRNAseq. This subset of 5-HT neurons from each group reflected electrophysiologic properties consistent with data from the total populations recorded (Figures 2G–J) where CO_2_ sensitive neurons had a higher mean C.I. than insensitive neurons (169.1 ± 14.2 vs. 94.8 ± 2.4, respectively; *P* < 0.0001; Figure 2G), consistently showed reversible increases in FR (Figure 2H) or no change (Figure 2I), had lower FRs under basal and recovery conditions (Figure 2J) where basal FRs were predictive of 5-HT neuron chemosensitivity (Supplementary Figures 1D,E).

### Single Cell RNA Sequencing of Phenotypically Distinct Brainstem 5-HT Neurons

**FIGURE 3:**
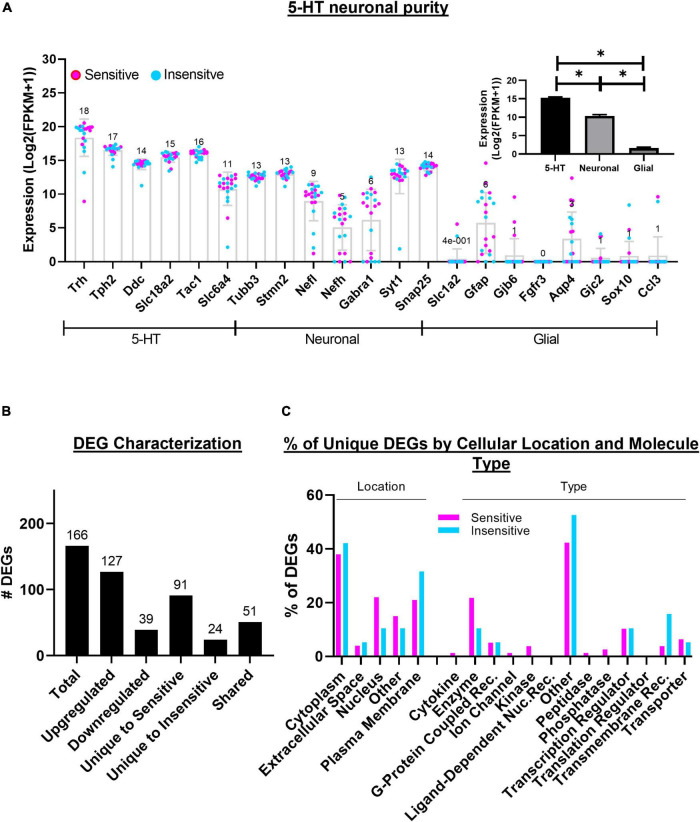
5-HT neuron RNA sequencing data are highly representative of 5-HT neurons. Expression [Log2(FPKM + 1)] of 5-HT, neuronal, and glial cell markers are indicated for each of the 21 neuron cDNA libraries that were sequenced **(A)** and 5-HT neuronal purity is statistically significant [**(A)**, inset]. Number and characteristics of differentially expressed genes (DEGs) among sequenced cells **(B)**. Subcellular location and molecular types represented across the 166 DEGs **(C)**. One-way ANOVA (*P* < 0.05) with Sidak’s *post hoc* tests **(A)**. *P < 0.0001.

Sequenced cDNA libraries from the recorded 5-HT neurons (*n* = 21) yielded a high-quality read summary (Table 1). Among the CO_2_ sensitive neurons, on average there were 5.690 million bases, over 46 million reads, over 82% of all bases had a Phred-like quality score of 30 or greater (higher indicates less probability of incorrectly identified base, e.g., Q30, 1 in 1,000 incorrect), an average quality score of 32.7, over 36 million of reads were mapped (∼75% of all reads), and 8,583 genes detected (Table 1A). Among the CO_2_ insensitive neurons, on average there were 6.090 million bases, over 50 million reads, over 82% of all bases had a Phred-like quality score of 30 or greater, an average quality score of 32.6, over 36 million of reads were mapped (∼75% of all reads), and 7,230 genes detected (Table 1B). We found higher normalized expression of canonical serotonergic genes compared to pan-neuronal and glial genes (15.26 ± 0.25, 10.34 ± 0.35, and 1.60 ± 0.25, respectively; *P* < 0.0001 for each comparison; Figure 3A inset). Therefore, the sequencing data is of high quality and contain high expression of 5-HT and neuronal markers with little evidence of contamination by local glia in 5-HT neurons.

**TABLE 1:** RNA sequencing read summary report for CO_2_ sensitive **(A)** and insensitive 5-HT neurons **(B)**.

Sample	Yield (million bases)	# Reads	% of ≥Q30 bases	Mean quality score	% Mapping rate	Mapped reads	# Genes detected (FPKM > 0)
**(A) CO_2_ sensitive**							
SC13	5.218	42,922,904	88.7	33.9	92.6	39,742,317	10,902
SC103	5.646	47,022,204	72.8	30.9	47.3	22,232,098	5,273
SC105	5.229	42,982,748	72.3	30.8	57.6	24,762,361	6,081
SC11	7.488	61,281,176	75.7	31.2	67.7	41,487,356	9,219
SC18	4.778	39,455,552	87.5	33.6	83.4	32,898,039	10,111
SC34	5.224	42,985,338	89.7	34.1	92.6	39,791,527	8,764
SC36	7.583	62,381,672	89.2	34	92.4	57,609,474	10,625
SC37	6.956	57,192,126	88.7	33.9	93.3	53,348,815	10,018
SC7	4.371	36,831,074	76.5	31.7	55	20,253,408	4,959
SC8	8.035	66,232,110	88.8	33.9	89.1	58,999,564	7,855
SC88	2.06	17,003,940	76.8	31.8	67.4	11,460,656	10,602
**Average:**	**5.69**	**46,935,531**	**82.43**	**32.71**	**76.22**	**36,598,692**	**8,583**

**(B) CO_2_ insensitive**							

SC106	6.046	50,011,382	73.1	30.9	67.3	33,672,664	10,682
SC110	6.819	56,121,406	76.2	31.2	65.7	36,894,212	6,923
SC133	8.001	65,703,648	77.9	31.4	32.7	21,504,804	3,430
SC135	7.022	57,494,206	74.7	31.1	61.5	35,330,190	6,706
SC21	4.392	36,116,268	88	33.7	85.1	30,720,498	5,496
SC25	6.586	54,582,514	89.4	34	87.8	47,945,280	5,227
SC30	6.519	53,789,910	89.9	34.1	91.2	49,051,019	9,675
SC31	6.509	53,516,874	89.2	34	93.2	49,888,430	10,098
SC43	5.576	46,001,240	88.9	33.9	90.9	41,810,527	9,395
SC46	3.425	28,585,488	76.2	31.7	56.4	16,122,215	4,663
**Average:**	**6.09**	**50,192,294**	**82.35**	**32.6**	**73.18**	**36,293,984**	**7,230**

*From left to right: The sample ID, the number of bases from sequencing the cDNA libraries in millions, total number of reads, the percentage of read bases with a Phred-quality score of 30 or greater, the mean Phred-quality score, percentage of reads mapped, the total number of reads mapped, and number of genes detected. The last row indicates the average for each quantified column of information.*

There were 166 differentially expressed genes (DEGs) identified based on a *p*-value corrected for multiple comparisons (*q*-value) < 0.05 (Figure 3B), 127 of which had increased, and 39 had decreased expression levels in CO_2_ sensitive 5-HT neurons relative to insensitive 5-HT neurons. 91 genes were uniquely expressed in CO_2_ sensitive 5-HT neurons and 24 genes uniquely expressed in CO_2_ insensitive 5-HT neurons. 51 genes were expressed in at least 1 neuron from both phenotypes. Ingenuity Pathway Analysis identified “cytoplasm” as the most represented cellular location for the gene products of identified DEGs across the groups, where ∼20% of all DEGs were categorized to encode an “enzyme” and ∼40% of DEGS were classified in the “other” category (Figure 3C).

**FIGURE 4:**
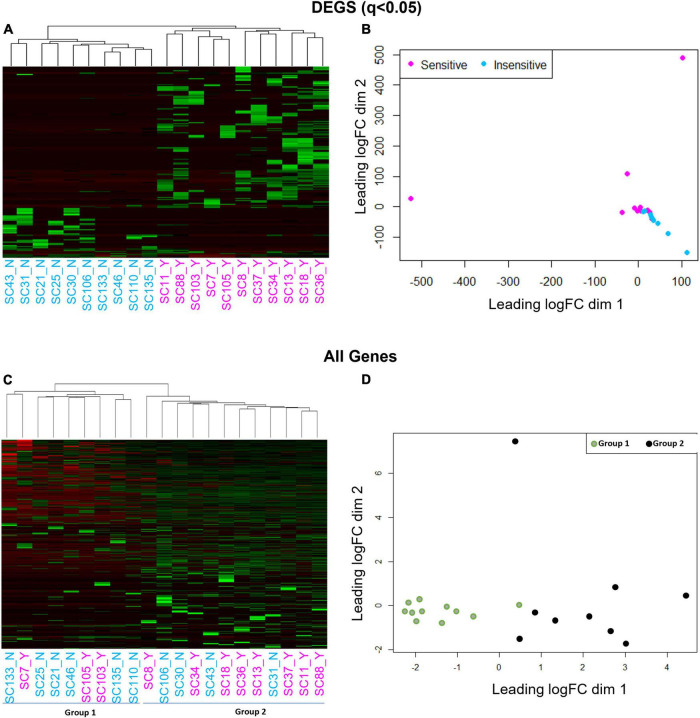
Hierarchical clustering **(A,C)** and multi-dimensional scaling [MDS; **(B,D)**] using all genes reveals a cell grouping unrelated to chemosensitivity versus grouping based on chemosensitivity with 166 DEGs. Hierarchical clustering and MDS using 166 DEGs result in distinct CO_2_ sensitive vs. insensitive 5-HT neuron grouping by hierarchical clustering and MDS **(A,B)**. Hierarchical clustering and MDS using all genes measured by scRNA-Seq results in two distinct cell groups un-related to CO_2_ chemosensitivity by hierarchical clustering and MDS **(C,D)**.

We next gauged the capacity for multi-dimensional scaling (MDS) and unbiased clustering analyses to segregate CO_2_ sensitive from insensitive 5-HT neurons based on transcriptomic data. Using the 166 DEGs led to visually distinct hierarchical clustering (Figure 4A) and MDS clusters (Figure 4B). However, this was not the case when we included all of the genes (DEGs and non-DEGs) detected from each 5-HT neuron. Omission of two cells (one with the highest dim 2 value and one with the lowest dim 1 value from Figure 4B) did not improve clustering patterns using either analysis (not shown). Rather, hierarchical clustering and MDS using all genes revealed two distinct groups of 5-HT neurons unrelated to previous chemosensitivity grouping criteria. Repeating the DEG analysis between these new groupings, Group 1 and 2, yielded over 3,000 DEGs (Figures 4C,D). Thus, *a priori* identification of the CO_2_ sensitivity phenotype using the patch-to-seq technique provided the necessary input to differentiate the 5-HT neurons and identify DEGs between CO_2_ sensitive and insensitive 5-HT neurons. These results allowed subsequent bioinformatic analyses to gauge predictive capacity of these DEGs in determining the CO_2_ sensitivity status of a 5-HT neuron.

### Pathway Analyses Suggest Biological Significance of Differentially Expressed Genes Across 5-HT Neuron Subpopulations

**FIGURE 5:**
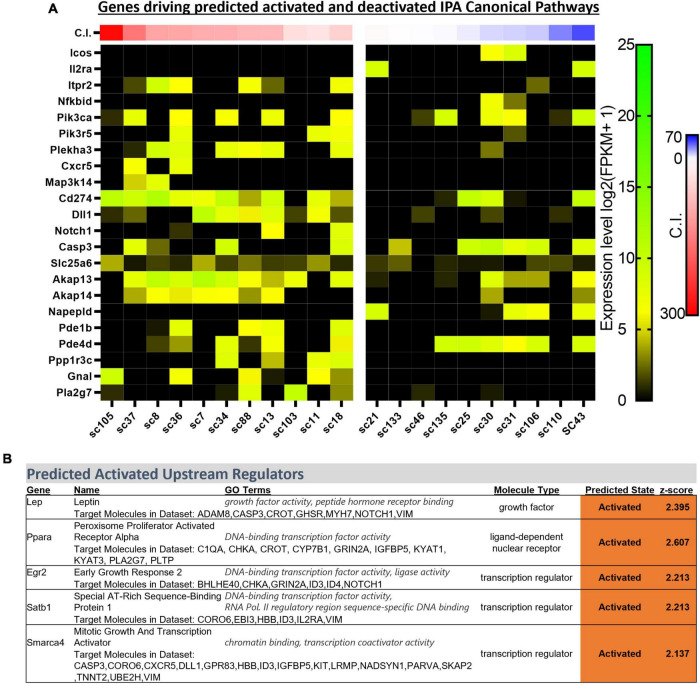
Heatmap of gene expression for each DEG contributing to activated z-scores across identified ingenuity pathway analysis (IPA) canonical pathways (indicated in the text) across all sequenced cells categorized by chemosensitivity index (C.I.) **(A)**. IPA predicted activated upstream regulators of 166 DEGs indicate *Egr2 and Lep*, among others, as activated in CO_2_ sensitive 5-HT neurons **(B)**.

Ingenuity Pathway Analyses can be a powerful approach for determining how multiple genes are linked within known biological processes and signaling pathways. To enhance the number of identified biological functions, canonical pathways, and predicted upstream regulators unique to CO_2_ sensitive neurons, the analyses from the previously identified 166 DEGs were expanded to include additional DEGs with more relaxed significance criteria (*p* < 0.01; Supplementary Figure 2). This generated significantly [−log (*p-*value) > 1.3] downregulated and upregulated canonical pathways (always CO_2_ sensitive relative to insensitive) as indicated by the predicted z-scores (Supplementary Figure 2B). Many of the identified pathways were related to immune system function (ICOS Signaling in T Helper Cells, NF-κB Activation by Viruses, Th1 Pathway, Induction of Apoptosis by HIV1, and CD40 signaling) with unknown biological relevance to 5-HT neuron function. However, many of the same DEGs contributed to significant z-scores greater than 2.0 across the identified canonical pathways (Figure 5A).

**FIGURE 6:**
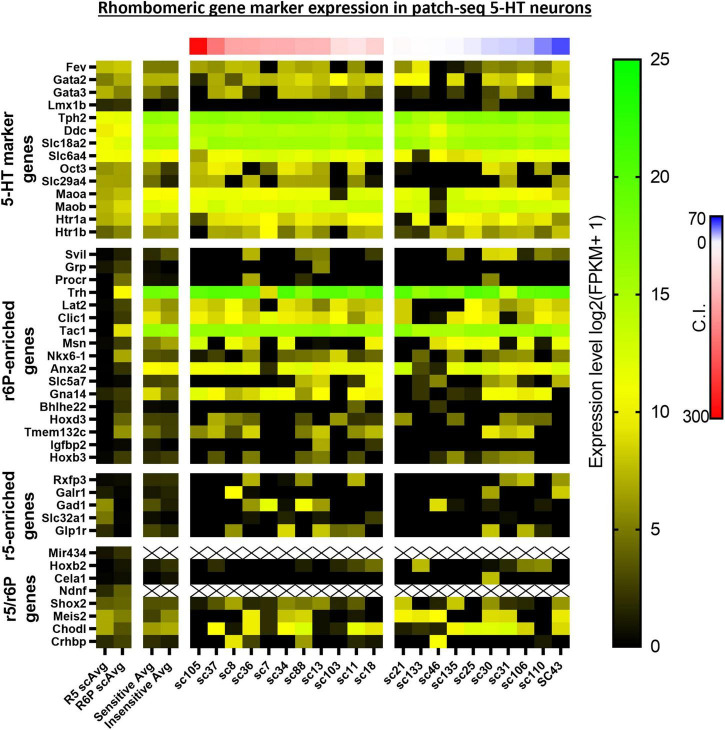
Heatmap of averaged single cell RNA Seq gene expression data derived from rhombomeric 5 (r5; i.e., CO_2_ sensitive 5-HT mouse neurons) and 6 (r6; i.e., CO_2_ insensitive 5-HT mouse neurons) in mice from a prior study by Okaty et al. (2015) compared to both averaged and single cell data of our 5-HT CO_2_ sensitive and insensitive rat neurons indicate differences in transcriptomic regulation of chemosensitivity between species. 5-HT marker genes (top horizontal panel) are enriched in both data sets. R6p-enriched genes (top middle horizontal panel) are highly expressed across all rat 5-HT neurons regardless of chemosensitivity while r5-enriched genes (lower middle horizontal panel) are undifferentiated between rat CO_2_ sensitive and insensitive 5-HT neurons. r5/r6p genes (bottom horizontal panel) are similarly expressed between CO_2_ sensitive and insensitive 5-HT rat neurons, consistent with the expression differences between R5 and R6p averages.

Upstream Regulator analyses identified 5 gene regulators predicted to be activated in CO_2_ sensitive neurons. Among these were 2 protein coding genes involved in metabolic regulation were predicted to be activated in CO_2_ sensitive neurons, *Lep* (Leptin; *z*-score: 2.395) and *Ppara* (Ppar alpha; *z*-score: 2.607; Figure 5B). These data suggest that CO_2_ sensitive 5-HT neurons exhibit increased activation of genes responsive to leptin, which to our understanding has not previously been linked to CO_2_ sensitive 5-HT raphe neurons. Furthermore, *Egr2* (*z*-score: 2.213), which is highly relevant given prior reports that mature CO_2_ sensitive 5-HT neurons are derived from 5-HT neurons that expressed *Egr2* (and *Pet*-*1* which is also known as *Fev*) during embryonic development (Brust et al., 2014). Prior fate mapping experiments in mice determined 5-HT neurons arise from unique cell lineages distinguished by the embryological expression of unique transcription factors. Specifically, rhombomere 5 (r5) and rhombomere r6p (posterior) give rise to the 5-HT neurons in the raphe magnus and obscurus, respectively. Electrophysiologic recordings in postnatal (P25-32) mice revealed the majority of r5 5-HT neurons were CO_2_ sensitive while most r6p 5-HT neurons were CO_2_ insensitive and that r5 and r6p neurons express distinct gene expression markers (Brust et al., 2014; Okaty et al., 2015). Whether r5 and r6p enriched genes had utility to distinguish the current rat neurons, and thus gauge if they might be embryologically determined, was assessed next. Expression of general 5-HT specific marker genes were consistent between the two data sets suggesting general similarities between mouse and rat 5-HT neurons (Figure 6). However, expression levels of the rat genes associated with R6P, R5, or r5/R6P enriched mouse genes did not cause any noticeable phenotypic separations (Figure 6). Rather, all the rat 5-HT neurons had similar gene expression levels as R6P-enriched genes. These data suggest that embryological gene markers may represent more anatomical separations of CO_2_ sensitive and insensitive 5-HT neurons and therefore most CO_2_ sensitive neurons are found in the r5 derived lineage, but not all. Thus, the current data represent a more specific transcriptional comparison between CO_2_ sensitive and insensitive 5-HT neurons because they were postnatally separated based on chemosensitivity and not based on embryological gene expression patterns. Therefore, a series of manual gene analyses and application of machine learning algorithms were employed to identify the most useful gene markers of CO_2_ sensitive and insensitive 5-HT rat neurons.

### Supervised Machine Learning Identifies Candidate Gene Markers of 5-HT CO_2_ Sensitivity

**FIGURE 7:**
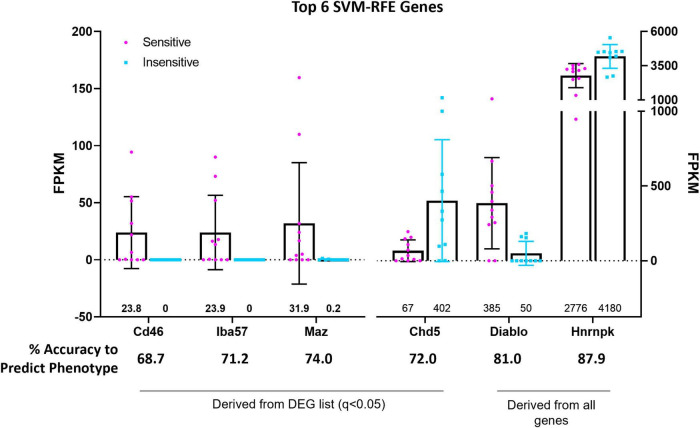
Support vector machine, recursive feature elimination (SVM-RFE) analyses identified the genes *CD46*, *Iba57*, *Chd5*, and *Maz* amongst 166 DEGs and the genes *Hnrnpk* and *Diablo* among all identified and sequenced genes as most accurate predictors of 5-HT neuron CO_2_ sensitivity (sensitive vs. insensitive).

Support vector machine-recursive feature elimination (see section “Materials and Methods” for details) bioinformatic analyses were used to refine and validate the predictive capability of select DEGs with greatest probabilities of differentiating between 5-HT CO_2_ sensitive and insensitive 5-HT neurons. The SVM-RFE performed on the 166 DEG list resulted in 4 candidate genes (*CD46*, *Iba57*, *Maz*, and *Chd5*), that each had high predictive accuracies (68.7, 71.2, 72.0, and 74.0%, respectively) in determining whether a 5-HT neuron is CO_2_ sensitive or insensitive (Figure 7). *CD46*, *Iba57*, and *Maz*, were expressed only in CO_2_ sensitive neurons while *Chd5* had expression in both types of neurons. *CD46* and *Iba57* were the only two genes from the SVM-RFE analysis that were identified in the top 10 CO2-enriched genes among the 166 DEGs (no SVM-RFE genes were observed in the top 10 DEGs or top 10 CO_2_-insensitive genes; Supplementary Figure 3). Two selected genes, *Cd46* and *Iba57*, had gene expression observed throughout entire brainstem tissue sections inclusive of the raphe magnus (RMg) using RNAScope. Whether Cd46 and Iba57 are strictly co-localized to neurons was not assessed, but they were indeed co-expressed in serotonergic (Tph2+) neurons, consistent with RNA-Seq derived data (Supplementary Figures 4, 5). Performing SVM-RFE using all identified genes resulted in 2 candidate genes, *Hnrnpk* and *Diablo*, which have predictive accuracies of 81.0 and 87.9%, respectively.

### Logistic and Linear Regression Between Candidate Gene Expression and Chemosensitivity Index

**FIGURE 8:**
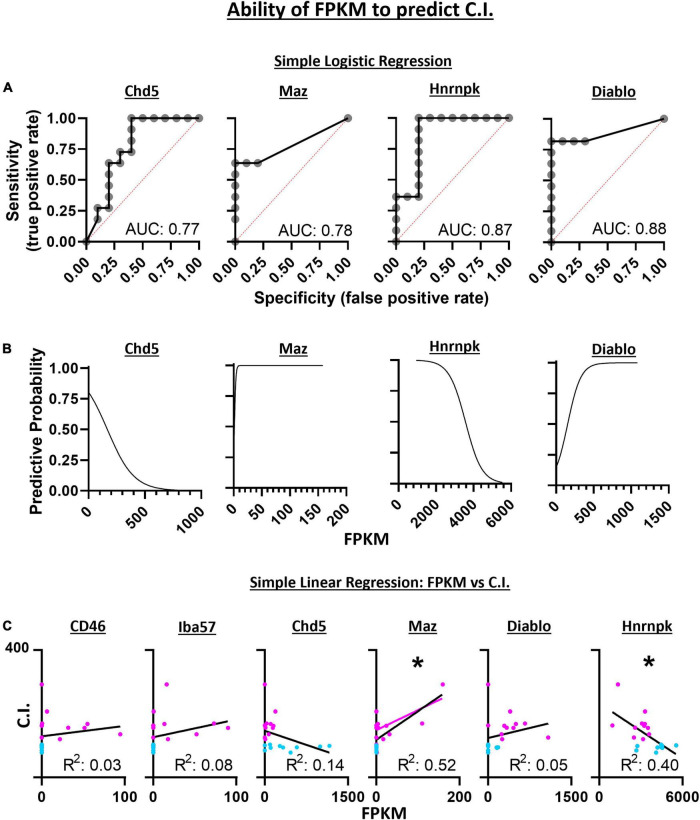
Simple logistic regression indicates the expression level (FPKM) of *Chd5*, *Maz*, *Hnrnpk*, and *Diablo* have high sensitivity and specificity with predictive accuracies (area under the curve) similar to the SVM-RFE analyses **(A)**. With increasing gene expression (FPKM), the predictive accuracy for assessing the chemosensitivity each neuron decreases for *Chd5* and *Hnrnpk* but the accuracy increases within increasing *Maz* and *Diablo* gene expression **(B)**. The expression levels of *Maz* and *Hnrnpk* but not *CD46*, *Iba57*, *Chd5*, or *Diablo* predict the degree of chemosensitivity of each 5-HT neuron assess by simple linear regression **(C)**. *Maz* and *Hnrnpk* have significant *R*^2^ values indicating their FPKM values account for a significant degree of the C.I. variability with a significant positive and negative Pearson *r* correlation, respectively. *P < 0.002; Pearson *r* correlation.

Diagnostic ability of the six candidate genes derived from SVM-RFE was assessed using logistic regression and a receiver operator characteristic (ROC) curve. These methods measured the associated degrees of specificity (true negative rate) and sensitivity (true positive rate) in assessing whether a 5-HT neuron is CO_2_ sensitive or insensitive. The area under the curve (AUC) for *Chd5* (0.77 ± 0.11; *p* = 0.0346), *Maz* (0.78 ± 0.11; *p* = 0.0290), *Hnrnpk* (0.87 ± 0.88; *p* = 0.0039) and *Diablo* (0.88 ± 0.084; *p* = 0.0031) were comparable to the predictive accuracies measured using SVM-RFE (Figure 8A). FPKM values were plotted against ROC derived predictive probabilities to determine if greater gene expression improved or detracted from the predictive accuracies for each gene (Figure 8B). Whereas increasing *Chd5* and *Hnrnpk* gene expression reduces predictive probability, increasing *Maz* and *Diablo* gene expression increases predictive probabilities. Thus, *Chd5* and *Hnrnpk* are better suited to identify CO_2_ insensitive 5-HT neurons because they are expressed at higher levels in CO_2_ insensitive vs. sensitive 5-HT neurons. *Maz* and *Diablo* gene expressions are better suited to identify CO_2_ sensitive 5-HT neurons because they are expressed at higher levels in CO_2_ sensitive than insensitive 5-HT neurons Logistic regression, ROC curves, and the AUC could not be calculated for *CD46* and *Iba57* because of perfect separation of gene expression (i.e., only CO_2_ sensitive 5-HT neurons expressed *CD46* and *Iba57*). Such perfect separation indicates that *CD46* and *Iba57* have high predictive ability to determine if 5-HT neurons are CO_2_ sensitive.

Linear regression was used to assess the ability for gene expression levels to predict the *degree* of chemosensitivity. *Maz* was found to have a significant best fit line (*R*^2^ = 0.52; *P* = 0.0002) as was *Hnrnpk* (*R*^2^ = 0.40; *P* = 0.0021) indicating *Maz* and *Hnrnpk* gene expression can account for a large degree of C.I. variability. A two-tailed Pearson *r* correlation analysis found *Maz* to have a significant positive correlation (*r* = 0.72; *P* = 0.0002) and *Hnrnpk* to have a significant negative correlation (*r* = −0.63; *P* = 0.0021) between gene expression and C.I. (Figure 8C).

## Discussion

In this study, we used the patch-to-seq technique and a combination of supervised, unsupervised, and software-based bioinformatic analysis techniques to identify functional and transcriptomic predictors of CO_2_ sensitive 5-HT neurons in the brainstem. We determined favorable diagnostic probabilities of using baseline firing rate as a predictor of 5-HT neuron chemosensitivity, suggesting low baseline firing rates may be a functional marker of CO_2_ sensitive 5-HT neurons. We identified 166 DEGs among CO_2_ sensitive and insensitive 5-HT neurons validating the original hypothesis that these subpopulations of 5-HT neurons are transcriptionally distinct. Bioinformatic pathway analyses revealed functional pathways not previously associated with neuronal chemosensitivity including a possible role of leptin and early growth response factor 2 (*Egr2*) in mediating gene expression in CO_2_ sensitive but not CO_2_ insensitive 5-HT neurons. Embryologic expression of *Egr2* gives rise to 5-HT CO_2_ sensitive neurons (Brust et al., 2014) and facilitate the adult hypercapnic ventilatory response (dependent on cellular CO_2_ chemoreception) (Ray et al., 2013). Additional bioinformatic analyses identified 6 novel candidate gene markers of CO_2_ sensitive 5-HT neurons that have a high accuracy (68.7–87.9%) in predicting if a 5-HT neuron is CO_2_ sensitive or insensitive, where two candidate gene markers, *CD46* and *Iba57*, were confirmed expressed in 5-HT neurons by RNA scope.

### Transcriptional Regulation in 5-HT CO_2_ Sensitive Neurons

The rationale that functionally distinct subpopulations are reflected by distinct transcriptomes stems from prior studies where intersectional fate mapping and single cell RNA sequencing clearly delineate rhombomere 5 (R5)-derived 5-HT neurons from the posterior rhombomere 6 (R6P) subgroups (Okaty et al., 2015). The r5 lineage gives rise to the 5-HT neurons that populate raphe magnus, which contained the overwhelming majority of CO_2_ sensitive 5-HT neurons compared to the raphe obscurus (derived from the r6 lineage). Indeed, the identification of 166 DEGs among CO_2_ sensitive and insensitive 5-HT neurons support the hypothesis that these functionally distinct 5-HT neuron subpopulations are transcriptionally distinct. Additionally, transcriptional analyses using unsupervised MDS and hierarchical clustering accurately grouped neurons by CO_2_ sensitivity. However, using more relaxed significance criteria and removing outlier cells to enhance pathway analyses led to more than 3000 DEGs that when used with the same unsupervised MDS and hierarchical clustering analyses, failed to group cells based on CO_2_ sensitivity. Instead, two other distinct groups of cells were observed indicating that a phenotype other than CO_2_ sensitivity is more distinguishable at the transcriptional level in these 5-HT neurons. Thus, the differences in cell grouping resulting from these two sets of analyses highlight the utility of the patch-seq technique in coupling cellular phenotypes to group cells to identify transcriptional differences that may underly functionally distinct cells.

We also reasoned that gene expression differences measured between CO_2_ sensitive and insensitive 5-HT neurons here might represent specific rhombomeric lineages. Previous studies show that rhombomeric origin was predictive of CO_2_ sensitivity in 5-HT neurons. Here we asked if functional distinctions among brainstem 5-HT neurons in rats aligned with gene markers of the R5 and R6P lineages in mice. While the R5 and R6P gene markers did not provide utility in distinguishing CO_2_ sensitive from insensitive 5-HT rat neurons, there were similar expression patterns of canonical 5-HT markers between datasets. Note, canonical 5-HT markers are similarly expressed across all 5-HT neurons despite lineage and/or phenotype. Despite confirming similar expression patterns of 5-HT neuron gene markers amongst both data sets, rhombomere-specific gene markers do not distinguish CO_2_ sensitive from insensitive 5-HT rat neurons. Thus, rhombomeric gene markers, while providing predicative power in whether a 5-HT neuron will develop into a CO_2_ sensitive or insensitive 5-HT neuron is clear from prior studies, the same genes do not appear to have a role in driving functional differences in postnatal 5-HT neurons. While this conclusion assumes rhombomere gene markers are similar in mice and rats, there is support that this may be the case given the very similar expression levels and profiles of the 5-HT marker genes across rat and mouse 5-HT neurons.

### Do the Differentially Expressed Genes Identified Functionally Contribute to Cellular 5-HT CO_2_ Sensitivity?

Our prior study identified specific pH sensitive potassium ion channels as putative mechanisms of cellular CO_2_ sensitivity in 5-HT neurons (Puissant et al., 2017). However, the previous technique suffered from a lack of functional data and neuronal specificity due to incomplete cellular dissociation. Our current approach yielded highly specific 5-HT neuron contents and functional data, again highlighting a strength in the patch-seq approach. However, the data generated by the patch-to-seq data failed to identify differential expression of previously identified pH sensitive K channel genes among CO_2_ sensitive and insensitive 5-HT neurons. In addition, the DEGs identified herein do not obviously functionally contribute to this cellular property based on what is known about their protein function. Pathway analyses did yield novel information about gene sets that strongly associate with CO_2_ chemosensitivity, including immune-related signaling pathways as previously found using an alternative approach (Mouradian et al., 2016). Predicted upstream regulators of these immune-related pathways showed 5 genes that were all predicted to be activated in CO_2_ sensitive neurons whereas there were no upstream regulators (activated or deactivated) in CO_2_ insensitive neurons. These data further suggest CO_2_ sensitivity in 5-HT neurons is transcriptionally regulated by upstream regulators whose function involves immune related functions, but if or how these specific upstream regulators contribute to the cellular properties of chemosensitivity to CO_2_/pH remains unclear.

The transcription factor *Egr2* has key roles in fate determination of 5-HT neurons and 5-HT neurons with a history of *Egr2* expression have cellular CO_2_ responses (Brust et al., 2014; Okaty et al., 2015). Pharmacogenetic inhibition of neurons with a history of *Egr2* expression in adult mice inhibits the hypercapnic ventilatory response, further indicating that *Egr2* is a marker of central respiratory chemoreceptor neurons (Ray et al., 2013). Thus, *Egr2* could be an alternative marker to specifically distinguish CO_2_ sensitive from insensitive 5-HT neurons, but *Egr2* in these neurons may only be expressed during embryologic development given that *Egr2* gene expression was not detected in the sequencing data. Also identified as an upstream regulator with high activation in CO_2_ sensitive 5-HT neurons was leptin, a key regulator of energy homeostasis. This suggests that CO_2_ sensitive 5-HT neurons are differentially responsive to leptin signaling compared to CO_2_ insensitive 5-HT neurons. Given that leptin is a key metabolic regulator, it’s potential to influence CO_2_ sensitive 5-HT neuronal gene expression may provide a link between metabolic and respiratory control. Indeed, CO_2_ sensitivity at the whole-animal level, measured by the hypercapnic ventilatory response is impaired long-term (measured from ∼30 to 230 days of age) in leptin-deficient (ob/ob) mice which is improved 3 days after leptin administration directly to the CNS (via 4th ventricle) but not subcutaneously (Tankersley et al., 1996; Bassi et al., 2012, 2015). Also, direct delivery of leptin into the nucleus of the solitary tract in the brainstem, a key site for integration of peripheral chemosensory afferents, causes an increase in CO_2_ chemosensitivity in normal rats (Inyushkin et al., 2009). Together, these data highlight an important stimulatory effect of CNS but not peripheral leptin on CO_2_ chemoreception.; While long-term obesity from genetic leptin deficiency causes long-term reductions in CO_2_ hypercapnic ventilatory responses, whether CO_2_ sensitivity at the neuronal level, and specifically in 5-HT neurons, is affected due to sustained elevated leptin levels as occurs in obesity remains unknown. However, leptin does have roles in a variety of biological functions, including reproduction, angiogenesis, blood pressure, and immune functions, the latter of which are predicted to be differentially regulated in CO_2_ sensitive 5-HT neurons (Zhang et al., 2005). Together, the 166 DEGs are putative mechanisms contributing to intrinsic chemosensitivity in 5-HT neurons and/or are markers of CO_2_ sensitive and insensitive 5-HT neurons.

### Molecular and Histological Markers of CO_2_ Sensitive 5-HT Neurons

We reasoned that the most practical molecular and histological markers of CO_2_ sensitive 5-HT neurons are those most strongly expressed in one cell type and not the other. Results indicate markers of CO_2_ sensitive 5-HT neurons would be better suited than markers of CO_2_ insensitive 5-HT neurons. Thus, using a combination of manual and machine learning analyses, 2 genes were identified that follow this expression pattern from the DEG list, *CD46* and *Iba57*. Gene expression of *CD46* and *Iba57* are limited to CO_2_ sensitive 5-HT neurons. Selection of these genes as histological markers within 5-HT neurons is supported by their high accuracy to predict the CO_2_ sensitivity phenotype calculated by SVM-RFE. Though simple linear regression is unable to correlate the expression level with C.I. for either *CD46* or *Iba57* independently, the majority (8 of 11) of CO_2_ sensitive 5-HT neurons express one or both genes while neither of the genes are expressed in any CO_2_ insensitive 5-HT neuron, making this pair of gene markers a viable histologic screening tool for identifying CO_2_ sensitive 5-HT neurons. Indeed, RNAScope verified expression of *CD46* and *Iba57* in 5-HT neurons (it did not test for co-expression among other cell types), though RNAScope did not validate the binary expression pattern of *CD46*+ and *Iba57*+ as suggested by the high percentage of 5-HT neurons expressing *CD46* and *Iba57*. This discrepancy between the sequencing results and RNA Scope may reflect the inability for total cell isolation using the patch-seq technique while RNA Scope can probe the contents of entire cells.

### Electrophysiologic Properties of CO_2_ Sensitive and Insensitive 5-HT Neurons

Our electrophysiologic data show that CO_2_ sensitive neurons have an average baseline firing rate (0.70 Hz) lower than CO_2_ insensitive neurons (1.306 Hz). These results are consistent with prior results derived from the perfused, *in situ* brainstem preparation [0.82 Hz vs. 1.82 Hz, respectively (Iceman et al., 2013)], acute brainstem slices (∼0.50 Hz vs. ∼1.5 Hz) (Brust et al., 2014) and primary cultures (Wang et al., 2001). While lower firing rates have been shown to be a feature of CO_2_ sensitive 5-HT neurons (Brust et al., 2014), the diagnostic criteria for baseline firing rate to predict if a 5-HT neuron is chemosensitive was previously unknown. A logistic regression test demonstrated significant sensitivity (true positive rate) for determining if a 5-HT neuron is CO_2_ sensitive with high confidence (79% sensitivity) when the baseline firing rate criterion was <0.4481 Hz indicating a practical means to estimate if a 5-HT neuron is CO_2_ sensitive.

In summary, via the patch-seq technique, functionally distinct brainstem 5-HT neurons were identified to be transcriptionally distinct aiding subsequent identification of novel genes associated with cellular CO_2_/pH sensitive neurons. These differentially expressed genes may contribute to cellular CO_2_ chemoreception, serve as distinct cellular markers of CO_2_ sensitive 5-HT neurons, and may provide important links to the 5-HT system dysfunction associated with human pathologies such as SIDS and SUDEP.

## Data Availability Statement

The datasets presented in this study can be found in online repositories. The names of the repository/repositories and accession number(s) can be found below: https://www.ncbi.nlm.nih.gov/, PRJNA820863.

## Ethics Statement

The animal study was reviewed and approved by the Institutional Animal Care and Use Committee at the Medical College of Wisconsin.

## Author Contributions

GM and MH contributed to the conception and design of the study, and wrote the first draft of the manuscript. GM, PL, ED, and JLG contributed to statistical analyses. JG wrote RNA-Scope methods sections of the manuscript. All authors contributed to manuscript revision.

## Conflict of Interest

The authors declare that the research was conducted in the absence of any commercial or financial relationships that could be construed as a potential conflict of interest.

## Publisher’s Note

All claims expressed in this article are solely those of the authors and do not necessarily represent those of their affiliated organizations, or those of the publisher, the editors and the reviewers. Any product that may be evaluated in this article, or claim that may be made by its manufacturer, is not guaranteed or endorsed by the publisher.

## Footnotes

1
https://www.rdocumentation.org/packages/gplots/versions/3.1.1/topics/heatmap.2

## Supplementary Material

Supplementary Figure 1 Firing rates of all CO_2_ sensitive **(A)** and insensitive **(B)** 5-HT neurons. Average firing rates during baseline, CO_2_ challenge, and recovery compared among all CO_2_ sensitive and CO_2_ insensitive 5-HT neurons **(C)**. The sub-population of neurons used for scRNA-Seq had representative firing rate differences during baseline, CO_2_ challenges, and recovery albeit one CO2 insensitive cell had oddly very high firing rates **(D–F)**. Notably, baseline and recovery firing rates were lower in CO_2_ sensitive vs. insensitive 5-HT neurons while firing rate during the CO_2_ challenge was similar between CO_2_ sensitive and insensitive neurons. One-way repeated measures ANOVA with Sidak’s multiple comparisons test **(A,B,D,E)**. ANOVA results: Condition *p* < 0.0001 and Individual Cell *p* < 0.001 **(A)**; Condition and Individual Cell *p* < 0.0001 **(B)**; Condition *p* < 0.0001 and Individual Cell *p* = 0.001 **(D)**; Condition *p* = 0.0510 and Individual Cell *p* < 0.0001 **(E)**. Two-way repeated measures ANOVA with Sidak’s multiple comparisons test **(C,F)**. Condition *p* < 0.0001, Cell Phenotype *p* = 0.0040, and Interaction *p* < 0.0001 **(C)**; Condition *p* < 0.0001, Cell Phenotype *p* = 0.0379, and Interaction *p* < 0.0001 **(F)**. **p* < 0.05 and ns, not significant Simple logistic regression using all cell recordings **(G)** or recordings from only cells that were sequenced **(I)** indicates that baseline firing rate of 5-HT medullary raphe neurons has high sensitivity and specificity with predictive accuracies (area under the curve) in determining if a 5-HT neuron is CO_2_ sensitive. With lower baseline firing rates, the predictive accuracy for assessing the chemosensitivity phenotype of 5-HT neurons increases **(H,J)**. ***p* < 0.01, ****p* < 0.001, *****p* < 0.0001.

Supplementary Figure 2 Ingenuity pathway analysis (IPA) canonical pathways significantly represented, but not predicted to be activated, by the 166 differentially expressed genes between CO_2_ sensitive and insensitive 5-HT neurons determined by *q* < 0.05 **(A)**. IPA canonical pathways significantly predicted to be down regulated (blue) or upregulated (orange) in CO_2_ sensitive neurons versus CO_2_ insensitive 5-HT neurons **(B)**.

Supplementary Figure 3 Top 10 differentially expressed genes (DEGs, top panel), CO_2_ sensitive enriched genes (middle panel), and CO_2_ insensitive enriched genes (lower panel). Data indicate most regularity in gene expression for CO_2_ sensitive enriched genes compared to Top 10 DEGs and CO_2_ insensitive enriched genes (i.e., CO_2_ sensitive enriched genes are only expressed in CO_2_ sensitive neurons).

Supplementary Figure 4 RNAScope immunofluorescence validates co-expression of *CD46* in Tph2+ 5-HT neurons within the rat brainstem including the raphe magnus (RMg). 4x objective overlay z-stack image outlining an area of the RMg that includes Tph2-expressing (green) cells (brightness enhanced for visualization of tissue outline) **(A)**. Zoom in of RMg region outlined in **(A)** captured with a 20x objective showing Tph2+ (green; left), *CD46*+ (red; middle; contrast enhanced 40%), and an overlay (right; contrast enhanced by 40%) **(B)**. Inset in overlay is enhanced zoom of a single Tph2+ neuron identified by the white arrow. Percentage of RMg and all Tph2+ cell also expressing at least 1 *CD46* pixel **(C)**. **(A)** Scale bar = 100 μm, **(B)** scale bar = 50 μm.

Supplementary Figure 5 RNAScope immunofluorescence validates co-expression of *Iba57* in Tph2 + 5-HT neurons within the rat brainstem including the raphe magnus (RMg). 4x objective overlay z-stack image outlining an area of the RMg that includes Tph2-expressing (green) cells (brightness enhanced for visualization of tissue outline) **(A)**. Zoom in of RMg region outlined in **(A)** captured with a 20x objective showing Tph2+ (green; left), *Iba57*+ (red; middle; contrast enhanced by 40%), and an overlay (right; contrast enhanced by 40%). Inset in overlay is enhanced zoom of a single Tph2+ neuron identified by the white arrow **(B)**. Percentage of RMg and all Tph2+ cell also expressing at least 1 *Iba57* pixel **(C)**. **(A)** Scale bar = 100 μm, **(B)** scale bar = 50 μm.
