# Supplementary figures and images for: Patch-to-Seq and Transcriptomic Analyses Yield Molecular Markers of Functionally Distinct Brainstem Serotonin Neurons
Source: Front Synaptic Neurosci. 2022 Jun 30;14:910820. doi: 10.3389/fnsyn.2022.910820 (PMC9280690; doi:10.3389/fnsyn.2022.910820)

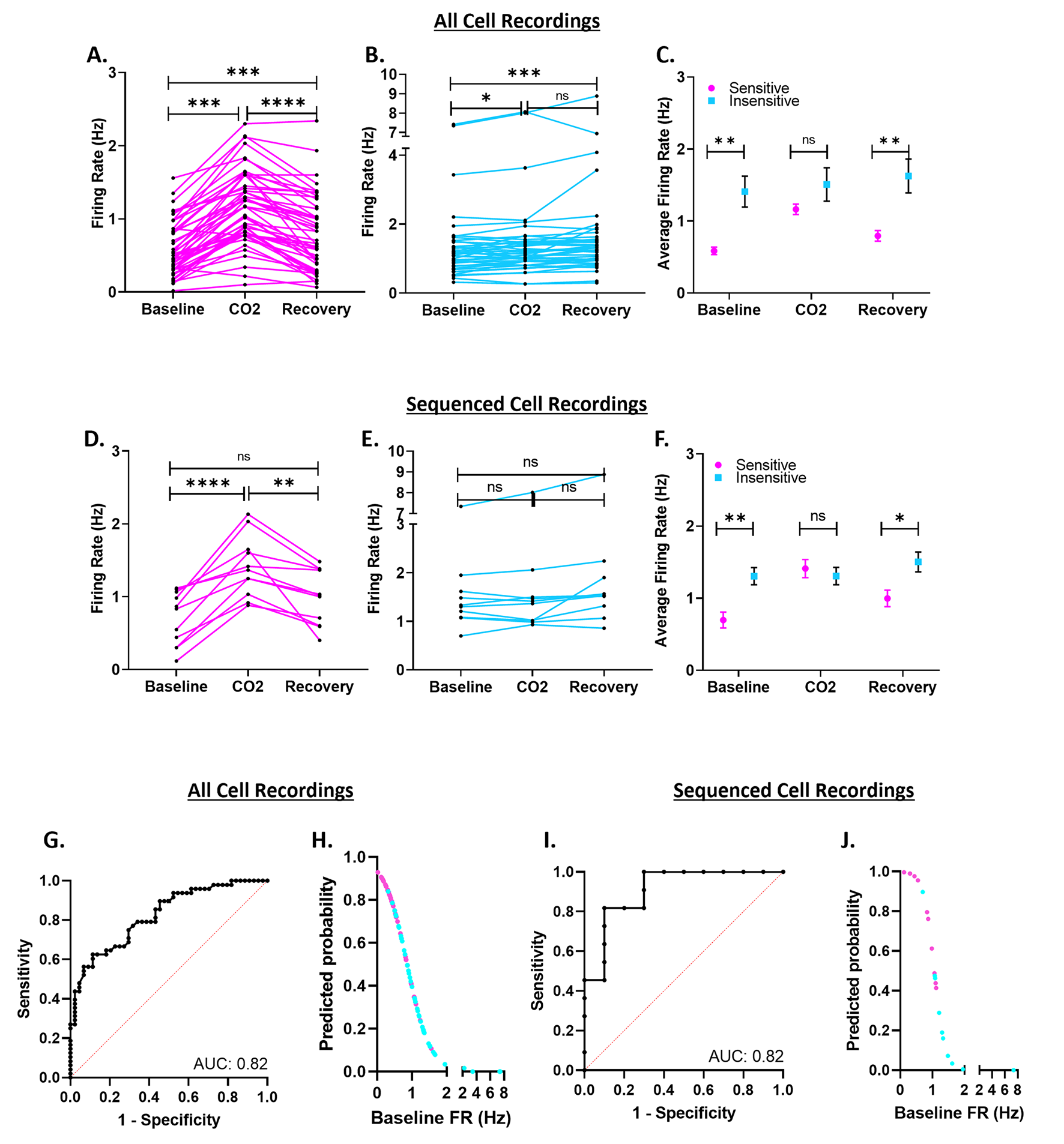

Supplement: Supplementary Figure 1 — Firing rates of all CO2 sensitive (A) and insensitive (B) 5-HT neurons. Average firing rates during baseline, CO2 challenge, and recovery compared among all CO2 sensitive and CO2 insensitive 5-HT neurons (C). The sub-population of neurons used for scRNA-Seq had representative firing rate differences during baseline, CO2 challenges, and recovery albeit one CO2 insensitive cell had oddly very high firing rates (D–F). Notably, baseline and recovery firing rates were lower in CO2 sensitive vs. insensitive 5-HT neurons while firing rate during the CO2 challenge was similar between CO2 sensitive and insensitive neurons. One-way repeated measures ANOVA with Sidak’s multiple comparisons test (A,B,D,E). ANOVA results: Condition p < 0.0001 and Individual Cell p < 0.001 (A); Condition and Individual Cell p < 0.0001 (B); Condition p < 0.0001 and Individual Cell p = 0.001 (D); Condition p = 0.0510 and Individual Cell p < 0.0001 (E). Two-way repeated measures ANOVA with Sidak’s multiple comparisons test (C,F). Condition p < 0.0001, Cell Phenotype p = 0.0040, and Interaction p < 0.0001 (C); Condition p < 0.0001, Cell Phenotype p = 0.0379, and Interaction p < 0.0001 (F). *p < 0.05 and ns, not significant Simple logistic regression using all cell recordings (G) or recordings from only cells that were sequenced (I) indicates that baseline firing rate of 5-HT medullary raphe neurons has high sensitivity and specificity with predictive accuracies (area under the curve) in determining if a 5-HT neuron is CO2 sensitive. With lower baseline firing rates, the predictive accuracy for assessing the chemosensitivity phenotype of 5-HT neurons increases (H,J). **p < 0.01, ***p < 0.001, ****p < 0.0001. [file Image_1.TIF]

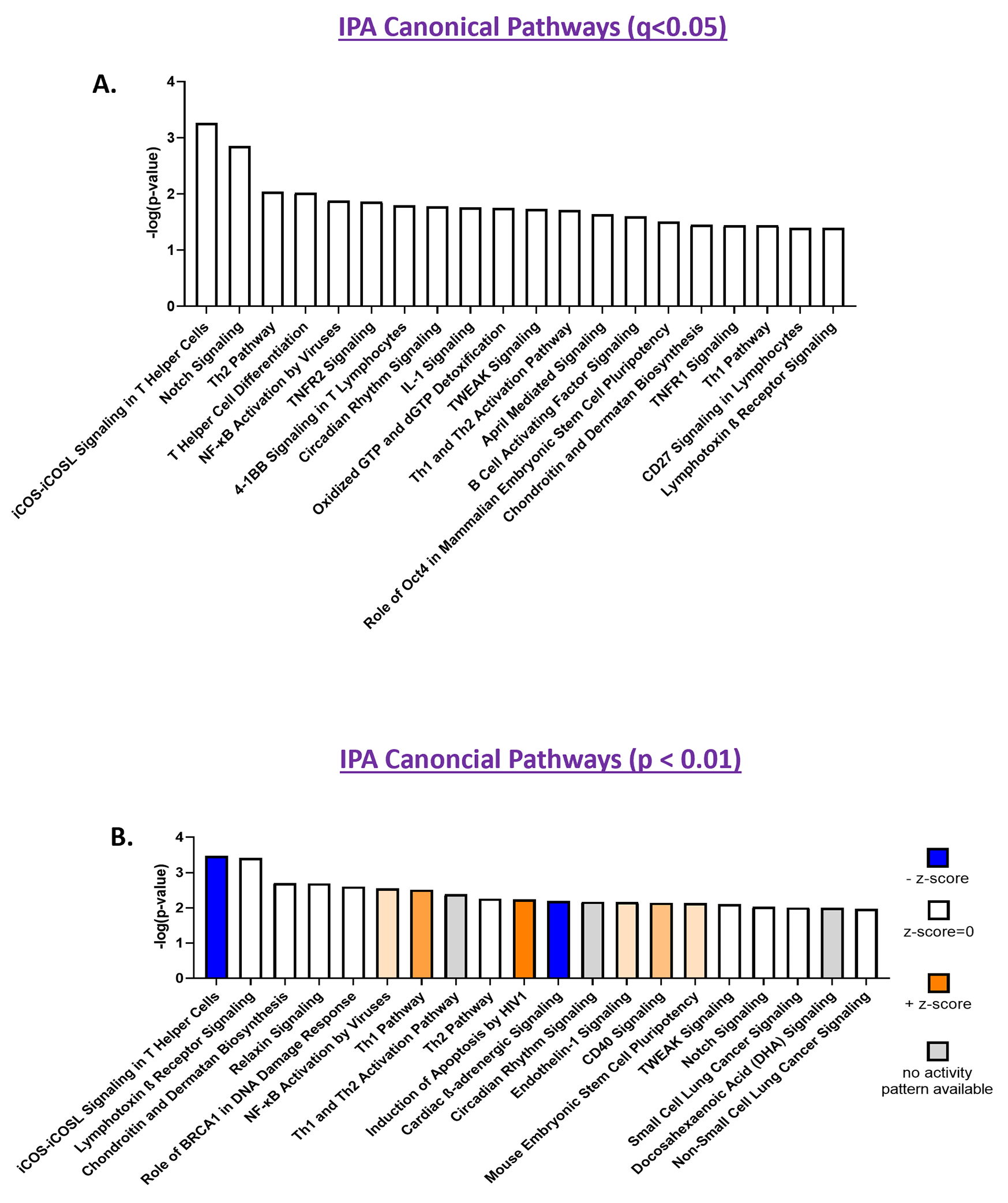

Supplement: Supplementary Figure 2 — Ingenuity pathway analysis (IPA) canonical pathways significantly represented, but not predicted to be activated, by the 166 differentially expressed genes between CO2 sensitive and insensitive 5-HT neurons determined by q < 0.05 (A). IPA canonical pathways significantly predicted to be down regulated (blue) or upregulated (orange) in CO2 sensitive neurons versus CO2 insensitive 5-HT neurons (B). [file Image_2.TIF]

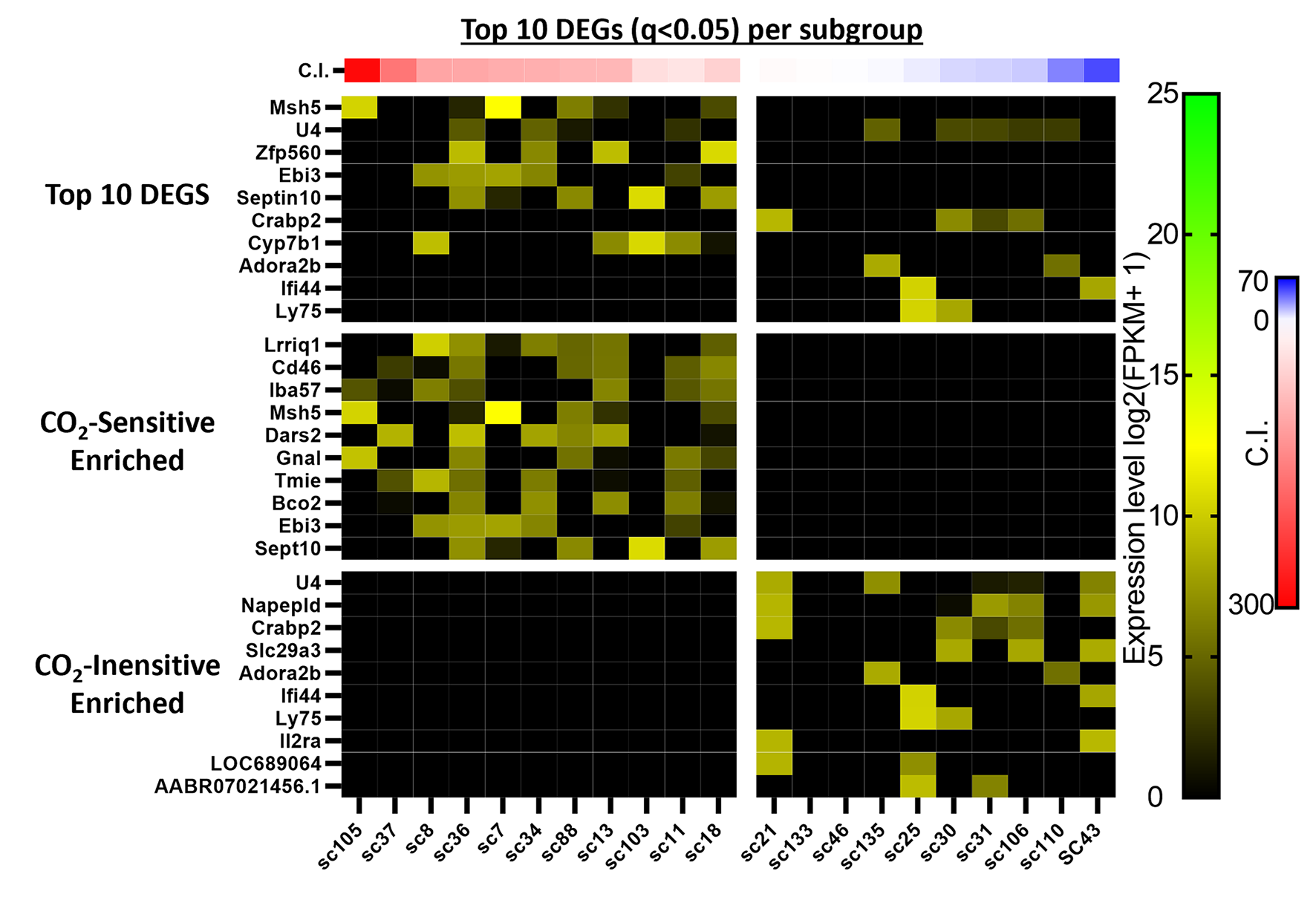

Supplement: Supplementary Figure 3 — Top 10 differentially expressed genes (DEGs, top panel), CO2 sensitive enriched genes (middle panel), and CO2 insensitive enriched genes (lower panel). Data indicate most regularity in gene expression for CO2 sensitive enriched genes compared to Top 10 DEGs and CO2 insensitive enriched genes (i.e., CO2 sensitive enriched genes are only expressed in CO2 sensitive neurons). [file Image_3.TIF]

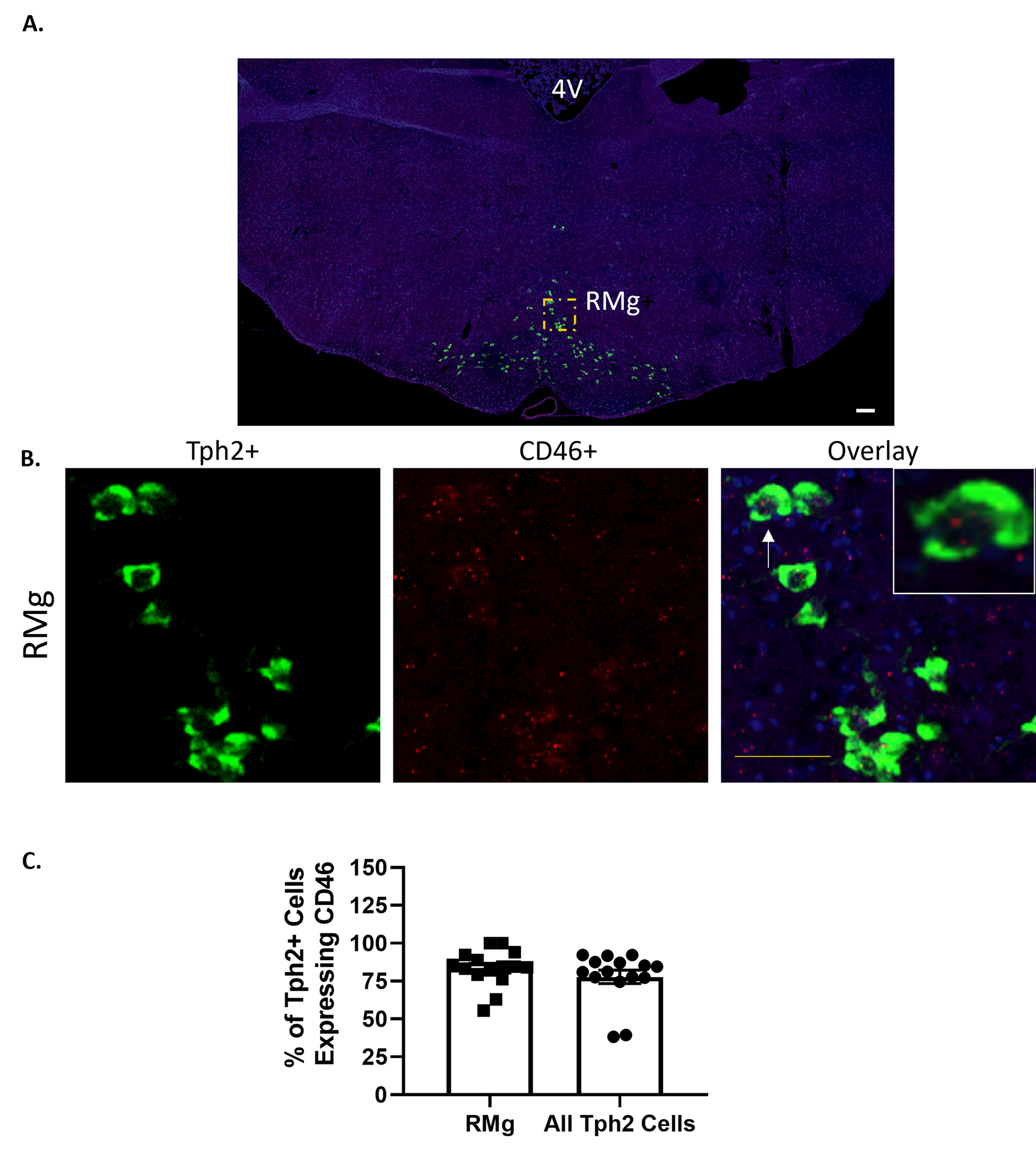

Supplement: Supplementary Figure 4 — RNAScope immunofluorescence validates co-expression of CD46 in Tph2+ 5-HT neurons within the rat brainstem including the raphe magnus (RMg). 4x objective overlay z-stack image outlining an area of the RMg that includes Tph2-expressing (green) cells (brightness enhanced for visualization of tissue outline) (A). Zoom in of RMg region outlined in (A) captured with a 20x objective showing Tph2+ (green; left), CD46+ (red; middle; contrast enhanced 40%), and an overlay (right; contrast enhanced by 40%) (B). Inset in overlay is enhanced zoom of a single Tph2+ neuron identified by the white arrow. Percentage of RMg and all Tph2+ cell also expressing at least 1 CD46 pixel (C). (A) Scale bar = 100 μm, (B) scale bar = 50 μm. [file Image_4.TIF]

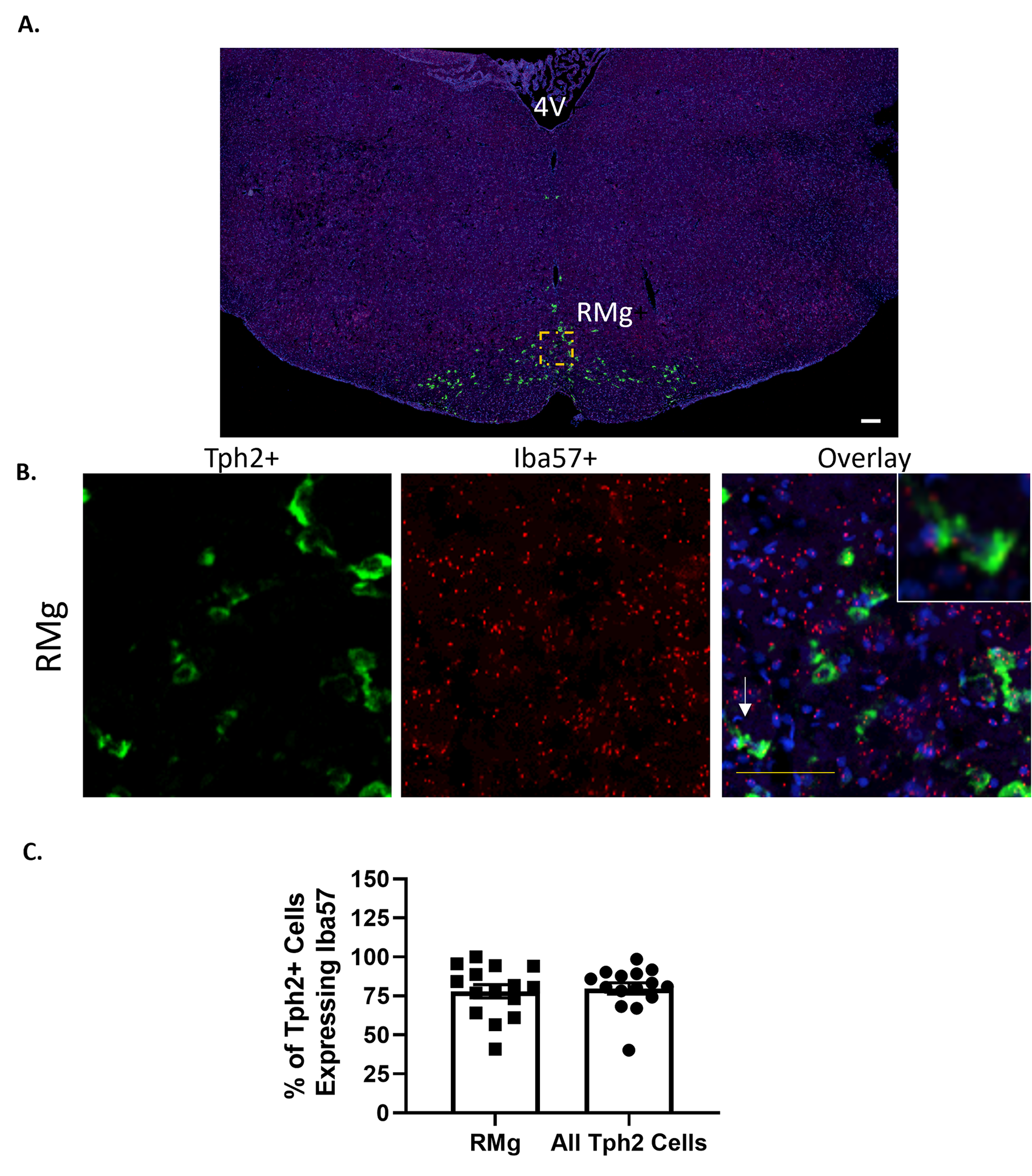

Supplement: Supplementary Figure 5 — RNAScope immunofluorescence validates co-expression of Iba57 in Tph2 + 5-HT neurons within the rat brainstem including the raphe magnus (RMg). 4x objective overlay z-stack image outlining an area of the RMg that includes Tph2-expressing (green) cells (brightness enhanced for visualization of tissue outline) (A). Zoom in of RMg region outlined in (A) captured with a 20x objective showing Tph2+ (green; left), Iba57+ (red; middle; contrast enhanced by 40%), and an overlay (right; contrast enhanced by 40%). Inset in overlay is enhanced zoom of a single Tph2+ neuron identified by the white arrow (B). Percentage of RMg and all Tph2+ cell also expressing at least 1 Iba57 pixel (C). (A) Scale bar = 100 μm, (B) scale bar = 50 μm. [file Image_5.TIF]
